# Quantitative Proteomic Analysis of the Response of Probiotic Putative *Lactococcus lactis* NCDO 2118 Strain to Different Oxygen Availability Under Temperature Variation

**DOI:** 10.3389/fmicb.2019.00759

**Published:** 2019-04-11

**Authors:** Wanderson Marques Da Silva, Leticia Castro Oliveira, Siomar Castro Soares, Cassiana Severiano Sousa, Guilherme Campos Tavares, Cristiana Perdigão Resende, Felipe Luis Pereira, Preetam Ghosh, Henrique Figueiredo, Vasco Azevedo

**Affiliations:** ^1^Departamento de Biologia Geral, Instituto de Ciências Biológicas, Universidade Federal de Minas Gerais, Belo Horizonte, Brazil; ^2^Departamento de Microbiologia, Imunologia e Parasitologia, Instituto de Ciências Biológicas e Naturais, Universidade Federal do Triangulo Mineiro, Uberaba, Brazil; ^3^AQUACEN, Escola de Veterinária, Universidade Federal de Minas Gerais, Belo Horizonte, Brazil; ^4^Department of Computer Science, Virginia Commonwealth University, Richmond, VA, United States

**Keywords:** *Lactococcus lactis*, bacterial proteomic, label-free proteomics, bacterial stress, bacterial physiology

## Abstract

*Lactococcus lactis* is a gram positive facultative anaerobe widely used in the dairy industry and human health. *L. lactis* subsp. *lactis* NCDO 2118 is a strain that exhibits anti-inflammatory and immunomodulatory properties. In this study, we applied a label-free shotgun proteomic approach to characterize and quantify the NCDO 2118 proteome in response to variations of temperature and oxygen bioavailability, which constitute the environmental conditions found by this bacterium during its passage through the host gastro-intestinal tract and in other industrial processes. From this proteomic analysis, a total of 1,284 non-redundant proteins of NCDO 2118 were characterized, which correspond to approximately 54% of its predicted proteome. Comparative proteomic analysis identified 149 and 136 proteins in anaerobic (30°C and 37°C) and non-aerated (30°C and 37°C) conditions, respectively. Our label-free proteomic analysis quantified a total of 1,239 proteins amongst which 161 proteins were statistically differentially expressed. Main differences were observed in cellular metabolism, stress response, transcription and proteins associated to cell wall. In addition, we identified six strain-specific proteins of NCDO 2118. Altogether, the results obtained in our study will help to improve the understanding about the factors related to both physiology and adaptive processes of *L. lactis* NCDO 2118 under changing environmental conditions.

## Introduction

*Lactococcus lactis* is a gram-positive lactic acid bacterium, and non-colonizing facultative anaerobe, that exhibits probiotic characteristic and can produce L-lactic acid through fermentative metabolism (Cavanagh et al., 2015). This bacterium exhibits simple metabolism and rapid growth and is used widely in food fermentation, amino acid production besides contributing to the preservation, flavor and texture characteristics of fermented products such as cheese and buttermilk. Due to its acceptance as a “GRAS” (Generally Recognized As Safe) and its capacity of surviving during the gut passage, *L. lactis* have been utilized as delivery systems for therapeutic molecules in the host gastro-intestinal tract (GIT) (Carvalho et al., 2017). The availability of whole genome sequences of several strains of *L. lactis* have contributed to both knowledge about the biology of this bacteria and the development of metabolic models, which provides information on its dynamic physiology (Dolatshahi et al., 2016; Oliveira et al., 2017; Yu et al., 2017).

*Lactococcus lactis* encounters environmental stresses, either in industrial processes or during passage through the GIT. Among the different stress types, temperature variation is a frequent condition encountered by this bacterium. For these reasons, some studies have evaluated both the physiological response and molecular mechanisms of *L. lactis* to thermal stress. The over-expression of the GroES-GroEL complex (Desmond et al., 2004) and DnaK (Al-Mahin et al., 2010) in *L. lactis* showed that these chaperones contribute significantly to its resistance to high temperature (Desmond et al., 2004). On the other hand, a genomic mutation generated in *L. lactis* through an adaptive laboratory evolution strategy allowed the isolation of a thermo-tolerant mutant strain (Chen et al., 2015). Other studies showed the importance of cold shock proteins in the adaptive response of *L. lactis* to low temperature as well as in cryoprotection (Wouters et al., 2000, 2001).

The oxygen availability is another environmental condition that *L. lactis* needs for survival, it must be able to adapt to high or low oxygen tensions, oxidative stress and oxygen limitation. Prior studies have shown that in respiratory conditions, oxygenation promotes alteration in its redox state and increases the NADH oxidase activity; consequently, the sugar fermentation happens to be a mixed fermentation leading to production of acetic acid, CO_2_, ethanol, acetoin, and lactic acid (Lopez de Felipe et al., 1997, 1998). On the other hand, the respiration stimulated by hemin improves its cellular growth as well as long term survival. In addition, under this condition, a metabolic alteration was observed along with an increase in the cell density in relation to anaerobic homolatic and aerobic heterolactic fermentation (Lan et al., 2006). The characterization of the *L. lactis* proteome under static, aerated, and respiration-permissive conditions (heme-dependent respiration) reveals that proteomic profiles of static and aeration cultures are indistinguishable. In turn, the comparative analysis between respiration and aeration conditions showed differences in proteins related to carbon metabolism and nitrogen metabolism (Vido et al., 2004).

NCDO 2118 is a strain of *L. lactis* subsp. *lactis* isolated from frozen peas that has been utilized as a model in some studies related to *L. lactis* metabolism (Garrigues et al., 1997, 2001). Interestingly, from such studies, it was shown that NCDO 2118 can biosynthesize GABA by glutamate decarboxylation, which is a molecule of nutraceutical interest capable of improving mood and muscle relaxation (Lapujade et al., 1998; Mazzoli et al., 2010). In addition, due to its ability to ferment xylose, a controlled xylose-inducible expression system was developed to target heterologous proteins to cytoplasm or extracellular medium (Miyoshi et al., 2004). Although a strain of environmental origin, studies recently showed that NCDO 2118 expresses recombinant proteins, such as: Hsp65 (Gomes-Santos et al., 2017), IL-10 (de Moreno de LeBlanc et al., 2016) and 15 LOX-1 (Saraiva et al., 2015; Carvalho et al., 2016) which are effective in treatment of inflammatory bowel diseases in experimental models. A study conducted by our group showed that NCDO 2118 exhibits anti-inflammatory and immunomodulatory activity against colitis in a murine model (Luerce et al., 2014). *In silico* analysis of the NCDO 2118 genome allowed for the identification of genes related to stress resistance, bacteriocins, adhesion-related and antibiotic resistance, that could contribute to its adaptive processes inside the host (Oliveira et al., 2014, 2017). More recently, our group performed a comparative proteomic study among NCDO 2118 and three biotechnological *L. lactis* strains, which allowed us to determine the *L. lactis* core-proteome as well as specific proteins that might contribute to the physiologic aspect of each strain (Silva et al., 2019).

In industrial process, lactic acid bacteria (LAB) need resistance to different types of technological stress during starter culture, production and storage of fermented foods as well as during the preparation of probiotic formulas. Posteriorly, probiotic LAB must survive and adapt to different stress conditions inside the GIT (Papadimitriou et al., 2016). To date, few studies have been conducted to evaluate the NCDO 2118 response to stress conditions. Our goal in this paper is to complement the results obtained in previous studies and expand our knowledge about the factors that might contribute to the adaptation of NCDO 2118 to stress conditions found by this bacteria inside the host GIT. To achieve this goal, we applied a Label-free proteomic approach to identify quantitative changes in the NCDO 2118 proteome in response to different oxygen availability and temperature variation.

## Materials and Methods

### Bacterial Strain, Growth Conditions and Preparation of Whole Bacterial Lysates

*L. lactis* subsp. *lactis* NCDO2118 was maintained in M-17 medium broth or agar (1.5%) (Difco, Franklin Lakes, NJ, United States) at 30°C. For proteomic analysis, overnight culture in M-17 supplemented with 0.5% (w/v) glucose (M17Glc) growth at 30°C without shaking were inoculated (1:100) in 50 mL of M-17 Glc fresh medium at 30°C or 37°C under non-aerated or anaerobic (anaerobic chamber) conditions, without shaking until it reached OD_600_ = 0.8. For anaerobic condition the culture flask was placed into a sealed anaerobic jar with a gas pack system (10% de CO_2_, 10% de H_2_, 80% de N_2_). All culture conditions were performed in biological triplicate. Cells were harvested by centrifugation 4,000 ×*g* for 10 min at 4°C. The cell pellets were washed twice with phosphate buffer saline (pH = 7) and then resuspended in 1 mL of lysis buffer [7 M urea, 2 M thiourea, 4% (w/v) CHAPS, and 1 M dithiothreitol (DTT)] and 10 μL of Protease Inhibitor Mix (GE Healthcare, Piscataway, NJ, United States) was added. The cells were broken by sonication at 5 × 1 min cycles on ice; the lysates were centrifuged at 14,000 ×*g* for 30 min at 4°C. Finally the protein extract were concentrated using a spin column with a 10 kDa threshold (Millipore, Billerica, MA, United States) and protein concentration was determined by Qubit 2.0 fluorometer (Invitrogen) using Qubit protein assay kit (Molecular Probes, Eugene, OR, United States) according to the manufacturer’s instructions.

### Protein Digestion

Proteins from each condition were denatured with *Rapi*GEST SF surfactant [0.1% (w/v) (Waters, Milford, CA, United States] at 60°C for 15 min. After reduction with DTT (10 mM) for 30 min at 60°C and alkylation with iodoacetamide (10 mM) (GE Healthcare), proteins were enzymatically digested with 1:50 (w/w) trypsin at 37 °C for 16 h (sequencing grade modified trypsin; Promega, Madison, WI, United States). The digestion process was stopped by adding 10 μl of 5% (v/v) Trifluoroacetic acid (TFA) (Fluka, Buchs, Germany). The peptide extracts were centrifuged at 21,900 × g for 30 min at 6°C, supernatants were transferred for Waters Total Recovery vials (Waters) and 1 N ammonium hydroxide was added. The samples were stored at -80°C until LC-MS analysis.

### nanoUPLC-HDMS^E^ Analysis

After tryptic digestion, a qualitative and quantitative proteomic analysis was carried out on the peptides, using a nanoACQUITY UPLC system (Nano Ultra Performance Liquid Chromatography Mass Spectrometry) (Waters Corp., Milford, MA, United States) coupled to Synapt G2-S high definition mass spectrometer (HDMS) (Waters Corp., Manchester, United Kingdom). Prior to proteomic analysis, stoichiometric measurements based on scouting runs of the integrated total ion account (TIC) were performed for all the samples; this analysis allows all samples to be injected with the same amount as well as standardized molar values across all conditions. The experiments were conducted using a 1-h reversed phase (RP) acetonitrile (0.1% v/v formic acid) gradient [7–40% (v/v)] at 500 nl/min on a nanoACQUITY UPLC 2D RP x RP Technology system (Waters). A nanoACQUITY UPLC High Strength Silica HSS T3 1.8 μm, 100 μm × 10 cm column (pH 3) was used in conjunction with a RP Acquity UPLC Nano Ease XBridge BEH130 C18 5 μm, 300 μm × 50 mm nanoflow column (pH 10). Typical on-column sample loads were 250 ng of the total protein digests for each of the five fractions that were generated for each sample (250 ng/fraction/load). For all measurements, the mass spectrometer was operated in resolution mode, with a typical effective m/z conjoined ion-mobility resolving power of at least 1.5 M FWHM, an ion mobility cell filled with helium gas, and a cross-section resolving power of at least 40 Ω/ΔΩ.

All analyses were performed using nano electrospray ionization in the positive ion mode nanoESI (+), and a Nano Lock Spray (Waters) ionization source. The lock mass channel was sampled every 30 s. The mass spectrometer was calibrated with a MS/MS spectrum of [Glu1]-fibrinopeptide B (Glu-Fib) human solution (100 fmol/μl) delivered through the reference sprayer of the Nano Lock Spray source. The double charged ion ([M + 2H]2+ = 785.8426) was used for initial single-point calibration, and MS/MS fragment ions of Glu-Fib were used to obtain the final instrument calibration. Multiplexed data-independent scanning with added specificity and selectivity of a non-linear “T-wave” ion mobility (HDMSE) experiments was performed using a Synapt G2-Si HDMS mass spectrometer (Waters). The radio frequency (RF) offset (MS profile) was adjusted so that the nanoUPLC-HDMSE data were effectively acquired from an *m/z* range of 400–2000 by MassLynx v.4.1 (Waters), which ensured that any masses observed in the high energy spectra of less than 400 m/z arose from dissociations in the collision cell.

### Processing of Mass Spectral Data

Protein identification and quantitative data packaging were generated using Progenesis QI for Proteomics (QIP) v.2.0 (Nonlinear Dynamics, Newcastle, United Kingdom). For peptide identification, data were searched against a *L. lactis* subsp *lactis* NCDO2118 database. The databases was processed in ProteinLynx Global Server (PLGS) v 3.0.2 tool (Waters) to create a reverse database and appended to the original database to assess the false positive rate of identification. The following parameters were used for peptide identification: maximum missed cleavages by trypsin (allowed up to one), variable carbamidomethyl of cysteine (fixed), acetyl N-terminal (variable), phosphoryl (variable), and oxidation (M) modifications; maximum protein mass: 600 kDa, Peptide tolerance: 10 ppm; fragment tolerance: 20ppm (Li et al., 2009). The protein-level quantitation was performed with Relative Quantitation using Hi-N algorithm, which is incorporated in Progenesis QIP. The search threshold for accepting each individual spectrum was set to the default value, with a false discovery rate (FDR) of 4%. The variability and quality of the proteomic data were analyzed through the distributions of peptide precursor and fragment error, peptide match distribution, drift time, and number of times that an identified protein appears on the biological replicates. The identified proteins were organized by the Progenesis QIP tool algorithm into a statistically significant list (ANOVA, *p-value* ≤ 0.05) corresponding to increased and decreased regulation ratios among the different groups. Only proteins with a differential expression ≥ 2-fold (log_2_ ratio ≥ 1 for proteins with higher abundance levels or log_2_ ratio ≤-1 for proteins with lower abundance levels) were considered.

### Bioinformatics Analysis

The identified proteins were analyzed using the following prediction tools. SurfG+ v1.0 (Barinov et al., 2009) was used to predict subcellular localization. To differentiate between the membrane and potentially surface exposed (PSE) proteins, this software utilizes the size of the membrane cell; in addition, SurfG+ contains additional tools such as *SignalP*, *LipoP* and *TMHMM* for the identification of motifs (Barinov et al., 2009); to predict gene ontology (GO) functional annotations, the COG database (Tatusov et al., 2001) was used. Pathway enrichment analysis was carried out using the Kyoto encyclopedia of genes and genomes (KEGG) database (Kanehisa and Goto, 2000). The OrthoMCL tool, which uses a Markov Cluster algorithm to group (putative) orthologs and paralogs (Li L. et al., 2003), was used to predict the exclusive ORF of *L. lactis* NCDO 2118. SecretomeP 2.0 server was used to predict proteins exported from non-classical systems (positive prediction scores greater than 0.5) (Bendtsen et al., 2005).

## Results and Discussion

### Global Proteomic Survey of NCDO2118 in Response to Different Growing Conditions

**FIGURE 1:**
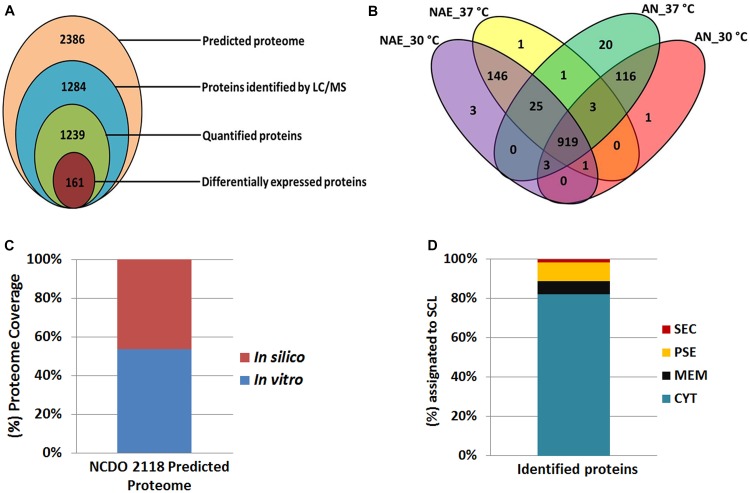
Proteomic data set. **(A)** Proteins identified and quantified by the label-free shotgun approach. **(B)** Venn diagram showing the overlap of the proteins identified among the conditions studied. **(C)** Correlation between proteomic results with *in silico* data of the NCDO 2118 proteome. **(D)** Subcellular localization (SCL) of the identified proteins by mass spectrometry.

In this study, we evaluated the *L. lactis* NCDO 2118 functional genome at the proteome level in response to no aerated (NAE) and anaerobic (AN) conditions exposed to 30°C and 37°C. NCDO 2118 was cultured in M17Glc medium (Supplementary File S1) and experimental stress conditions were performed in biological triplicates; proteins from total bacterial lysates were extracted and digested in solution. Then the resulting peptides were analyzed by LC/MS^E^ approach (Silva et al., 2006). Our proteomic analysis identified 1,138 proteins in AN condition with an average of six peptides per protein (Supplementary File S2, Table S1) and 1,139 proteins in NAE condition with an average of seven peptides per protein (Supplementary File S2, Table S2). In this analysis, we considered only proteins that were in agreement in two out of three replicates which resulted in a FDR of 0.92% and 0.76% in AN and NAE, respectively. All information about identified peptides for each protein, sequence coverage and information about the native peptides are available at Supplementary Files S3–S6. From this proteomic data set, we characterized a total of 1,284 non-redundant proteins of NCDO 2118, with at least one unique peptide (Figure 1A). Considering only proteins with at least two peptides per protein and proteins identified in at least two of the three biological replicates of each condition, our label-free proteomic analysis quantified a total of 1,239 proteins (Figure 1A). Differential expression was considered only for proteins that were significantly different with ANOVA *p*-value (*p* ≤ 0.05) and cut-off values of 2-fold change. Based on these parameters, 161 proteins were statistically differentially expressed (Figure 1A). Comparative analysis between NAE and AN conditions revealed a core proteome composed of 952 proteins (Figure 1B). Considering the exclusive proteome of each condition, 137 and 150 proteins were identified in the proteome of AN and NAE, respectively (Figure 1B and Supplementary File S2, Tables S3, S4).

**FIGURE 2:**
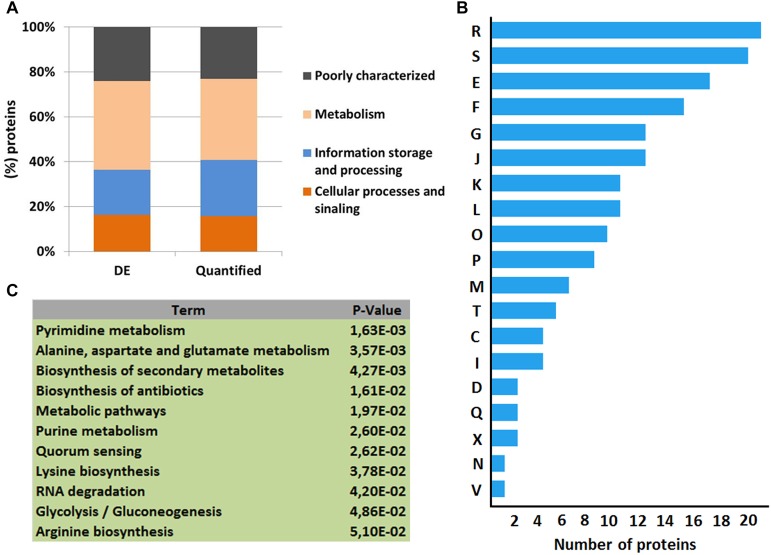
Functional analyses of the quantitative proteomic data set. **(A)** Clusters of orthologous groups of differentially expressed proteins (DE) and quantified proteins by LC–MS^E^ analysis. **(B)** Categorization of differentially expressed proteins in biological processes. [R] General function prediction only; [S] Function unknown; [E] Amino acid transport and metabolism; [F] Nucleotide transport and metabolism; [G] Carbohydrate transport and metabolism; [J] Translation, ribosomal structure and biogenesis; [K] Transcription; [L] Replication, recombination and repair; [O] Posttranslational modification, protein turnover, chaperones; [P] Inorganic ion transport and metabolism; [M] Cell wall/membrane/envelope biogenesis; [T] Signal transduction mechanisms; [C] Energy production and conversion; [I] Lipid transport and metabolism; [D] Cell cycle control, cell division, chromosome partitioning; [Q] Secondary metabolites biosynthesis, transport and catabolism; [X] Mobilome: prophages, transposons; [N] Cell motility; [V] Defense mechanisms. **(C)** KEGG pathways enrichment analysis of differentially expressed proteins.

When the total number of proteins identified by MS was overlapped against the NCDO 2118 genome, approximately 54% of its predicted proteome was characterized (Figure 1C). Using SurfG tool based analysis (Barinov et al., 2009), the identified proteins were grouped within the following categories: cytoplasmic (CYT), membrane (MEM), potentially surface-exposed (PSE) and secreted (SEC) (Figure 1D). To determine the functional characteristics of the quantified and differentially expressed proteins, we performed a Clusters of Orthologous Groups analysis (Tatusov et al., 2001). According to GO analysis, the proteins were organized by clusters of different orthologous groups: (i) metabolism, (ii) information storage and processing, (iii) cellular processes and signaling, and (iv) poorly characterized (Figure 2A). When evaluating the biological processes that comprise each category listed above, the differentially expressed proteins were distributed into 19 functional categories (Figure 2B). To obtain more functional information about differentially expressed proteins, KEGG pathway enrichment analyses was performed and according to Fisher’s exact test, eleven pathways had *p* ≤ 0.05 and were considered the most significant pathways (Figure 2C).

**FIGURE 3:**
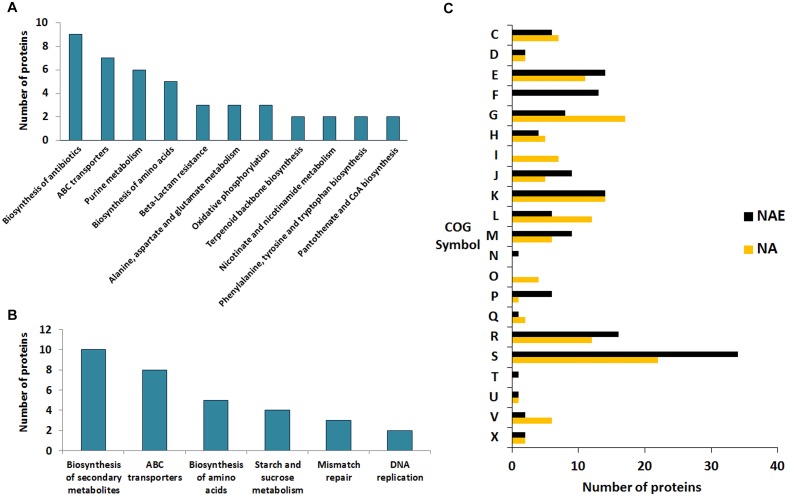
Comparative functional analyses between the proteins detected in NAE and AN conditions. **(A)** KEGG pathways enrichment analysis of the exclusive proteome of NAE condition. **(B)** KEGG pathways enrichment analysis of the exclusive proteome of AN condition. **(C)** Comparison between the biological processes detected in AN and NAE conditions. [C] Energy production and conversion; [D] Cell cycle control, cell division, chromosome partitioning; [E] Amino acid transport and metabolism; [F] Nucleotide transport and metabolism; [G] Carbohydrate transport and metabolism; [H] Coenzyme transport and metabolism; [I] Lipid transport and metabolism; [J] Translation, ribosomal structure and biogenesis; [K] Transcription; [L] Replication, recombination and repair; [M] Cell wall/membrane/envelope biogenesis; [N] Cell motility; [O] Posttranslational modification, protein turnover, chaperones; [P] Inorganic ion transport and metabolism; [Q] Secondary metabolites biosynthesis, transport and catabolism; [R] General function prediction only; [S] Function unknown; [T] Signal transduction mechanisms; [U] Intracellular trafficking, secretion, and vesicular transport; [V] Defense mechanisms; [X] Mobilome: prophages, transposons.

**FIGURE 4:**
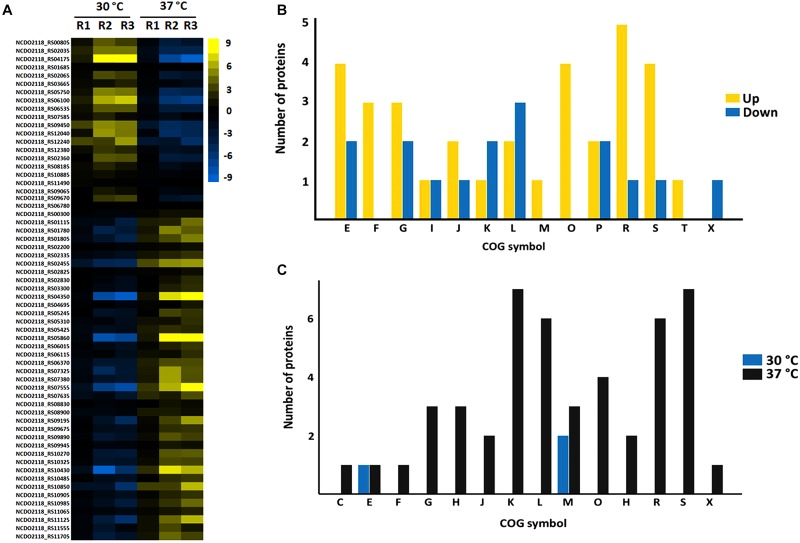
Label free proteomics of AN condition. **(A)** Differential proteome detected between 30°C vs. 37°C (the columns represent replicates of each condition), [Log (2) fold change, ANOVA *p* ≤ 0.05]. **(B)** Categorization of differentially expressed proteins between 37°C: 30°C in biological processes. [E] Amino acid transport and metabolism; [F] Nucleotide transport and metabolism; [G] Carbohydrate transport and metabolism; [I] Lipid transport and metabolism; [J] Translation, ribosomal structure and biogenesis; [K] Transcription; [L] Replication, recombination and repair; [M] Cell wall/membrane/envelope biogenesis; [O] Posttranslational modification, protein turnover, chaperones; [P] Inorganic ion transport and metabolism; [Q] Secondary metabolites biosynthesis, transport and catabolism; [R] General function prediction only; [S] Function unknown; [T] Signal transduction mechanisms; [X] Mobilome: prophages, transposons. **(C)** Functional classification of the proteins detected in the exclusive proteome of AN condition. [S] Function unknown; [G] Carbohydrate transport and metabolism; [K] Transcription; [L] Replication, recombination and repair; [R] General function prediction only; [E] Amino acid transport and metabolism; [C] Energy production and conversion; [I] Lipid transport and metabolism; [M] Cell wall/membrane/envelope biogenesis; [V] Defense mechanisms; [H] Coenzyme transport and metabolism; [J] Translation, ribosomal structure and biogenesis; [K] Transcription; [O] Posttranslational modification, protein turnover, chaperones; [D] Cell cycle control, cell division, chromosome partitioning; [Q] Secondary metabolites biosynthesis, transport and catabolism; [X] Mobilome: prophages, transposons; [P] Inorganic ion transport and metabolism; [U] Intracellular trafficking, secretion, and vesicular transport.

Proteins comprising the AN and NAE proteome were next individually evaluated; KEGG enrichment analysis showed that eleven and six pathways were statically significant in NAE and AN conditions, respectively (*p* ≤ 0.05) (Figure 3A,B). GO analysis showed that in NAE condition the majority of the proteins are related to amino acid transport and metabolism while in AN condition, the majority of the proteins are involved in carbohydrate transport and metabolism (Figure 3C). By evaluating the differential proteome of each condition, in AN condition (37°C vs. 30°C), we detected 66 proteins with significant statistical values (*p* ≤ 0.05) (Figure 4A and Supplementary File S2, Table S5). Considering the difference in their level of abundance (37°C: 30°C), 33 proteins were observed to be more induced while 17 were less induced. These proteins were next grouped into different biological processes, based on known or predicted functions; the more induced proteins were related mainly to amino acid metabolism and posttranslational modification, protein turnover, and chaperones. The majority of the less induced proteins were involved in replication, recombination and repair (Figure 4B). Similarly, evaluation of the exclusive proteome of 30°C (AN) and 37°C (AN) detected three and forty seven proteins, respectively (Supplementary File S2, Table S6). The proteins detected at 30°C are associated with amino acid transport and metabolism and cell wall/membrane/envelope biogenesis. Already, the 37°C exclusive proteome was composed mostly of proteins related to the transcriptional process (Figure 4C).

**FIGURE 5:**
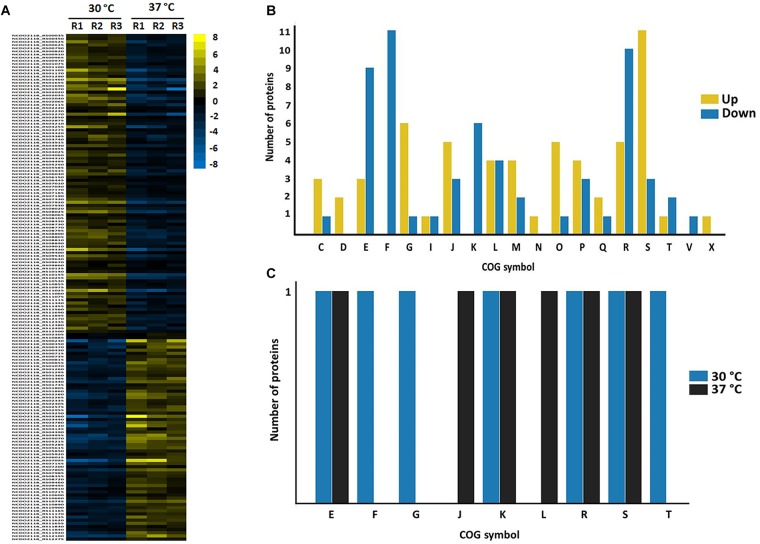
Label free proteomics of NAE condition. **(A)** Differentially expressed proteins between 30 vs. 37 °C (the columns represent replicates of each condition), [Log (2) fold change, ANOVA *p* ≤ 0.05]. **(B)** Categorization of differentially expressed proteins between 37°C: 30°C in biological processes. [C] Energy production and conversion; [D] Cell cycle control, cell division, chromosome partitioning; [E] Amino acid transport and metabolism; [F] Nucleotide transport and metabolism; [G] Carbohydrate transport and metabolism; [I] Lipid transport and metabolism; [J] Translation, ribosomal structure and biogenesis; [K] Transcription; [L] Replication, recombination and repair; [M] Cell wall/membrane/envelope biogenesis; [N] Cell motility; [O] Posttranslational modification, protein turnover, chaperones; [P] Inorganic ion transport and metabolism; [Q] Secondary metabolites biosynthesis, transport and catabolism; [R] General function prediction only; [S] Function unknown; [T] Signal transduction mechanisms; [U] Intracellular trafficking, secretion, and vesicular transport; [V] Defense mechanisms; [X] Mobilome: prophages, transposons. **(C)** Functional classification of the proteins detected in the exclusive proteome of NAE condition. [S] Function unknown; [R] General function prediction only; [K] Transcription; [E] Amino acid transport and metabolism; [F] Nucleotide transport and metabolism; [M] Cell wall/membrane/envelope biogenesis; [J] Cell wall/membrane/envelope biogenesis; [G] Carbohydrate transport and metabolism; [L] Replication, recombination and repair; [C] Energy production and conversion; [P] Inorganic ion transport and metabolism; [H] Coenzyme transport and metabolism; [V] Coenzyme transport and metabolism; [D] Cell cycle control, cell division, chromosome partitioning; [X] Mobilome: prophages, transposons; [Q] Secondary metabolites biosynthesis, transport and catabolism; [U] Intracellular trafficking, secretion, and vesicular transport; [N] Cell motility; [T] Signal transduction mechanisms.

In turn, in NAE condition (37°C vs. 30°C), 162 proteins presented significant statistical values (*p* ≤ 0.05) (Figure 5A and Supplementary File S2, Table S7). Among them, 117 proteins showed difference in their level of abundance (37°C: 30°C) wherein 58 proteins were more induced and 59 were less induced. The majority of the more induced proteins of known or predicted function were related to carbohydrate metabolism. On the other hand, the less induced proteins were involved mainly with nucleotide transport and metabolism (Figure 5B). In NAE, we also detected proteins exclusive to the proteome of 37°C (six proteins) and 30°C conditions (seven proteins) (Supplementary File S2, Table S8). The functional analyses grouped these proteins into different biological processes (Figure 5C).

### Stress Response Proteins

Our proteomic analysis detected a difference between the differentially expressed proteins of AN and AE conditions related to stress response, among which some are associated to oxidative stress. *L. lactis* is frequently confronted by oxidative stress both during the GIT passage and industrial processes. During the oxidative stress, reactive oxygen species (ROS) are produced which are highly toxic to cells. ROS promotes oxidation of cellular components, such as proteins, lipids and DNA (Cole, 2018; Martins et al., 2019). To combat this stress, *L. lactis* contains genes encoding antioxidant enzymes and enzymatic antioxidant systems, which can protect against ROS. Thiol peroxidase (Tpx) and Superoxide dismutase [Mn] (MnSOD) were detected as more induced at 37°C in both conditions. Tpx is an enzyme involved in the reduction process of H_2_O_2_ and other hydroperoxides, which contribute to resistance against oxidative stress (Flohé, 2016). Superoxide dismutase (SOD) are metalloenzymes described as a first line of defense among the antioxidant enzyme; this enzyme can rapidly catalyze superoxide (O_2_^-^) to hydrogen peroxide (H_2_O_2_) and dioxygen (O_2_) (Bresciani et al., 2015). *L. lactis* produces mainly Mn^2+^-binding SODs that is codified by the *sodA* gene (Sanders et al., 1995). An earlier study also showed that *L. lactis* requires MnSOD to protect against O_2_^-^ anion radicals. Additionally, it was shown that MnSOD exhibits highest activity under aerobic conditions, than anaerobic condition (Sanders et al., 1995). When we compared the expression levels of MnSOD between AN (37°C: 30°C) and NAE (37°C: 30°C) conditions, we observed fold change ratios of 1.52 and 1.35, respectively. In *Escherichia coli*, a transcriptome study showed the induction of *sodA* under anaerobic condition in response to H_2_O_2_ (Kang et al., 2013). Interestingly, the antioxidant activity in LAB is variable among strains under anaerobic or aerobic conditions, which might be either strain-specific or stress-specific (Li Y. et al., 2003; Ramesh et al., 2012).

Thioredoxin reductase (TrxB) was detected to be more abundant in AN condition (37°C: 30°C). TrxB belongs to the thioredoxin system, which is involved in thiol/disulfide balance in both prokaryotes and eukaryotes. A study showed that a *Lactobacillus casei* strain with defective trxB gene exhibits growth defects under anaerobic condition (Serata et al., 2012). Glutathione reductase (GR), an enzyme that catalyzes the reduction of glutathione, was detected only in the AN proteome. Due the capacity of *L. lactis* to accumulate high levels of intracellular glutathione, the latter might be utilized as an alternative mechanism to eliminate O_2_^-^ anion (Fahey et al., 1978; Li Y. et al., 2003). Hence, the identification of these proteins exhibits a set of potential molecules that may be crucial to combat the oxidative stress and maintain the redox homeostasis of NCDO 2118 during industrial processes or during the gut passage.

Chaperones are proteins that contribute also to prokaryotic resistance against different stress conditions. The chaperones GroES, GroEL, DnaJ and ClpB were induced in NAE condition at 37°C. In turn in AN condition (37°C: 30°C), only GrpE was detected to be more induced. GroEL-GroES and DnaK-DnaJ-GrpE complexes are the major chaperone systems in prokaryotes (Lund, 2001). These chaperone complexes, together with other chaperones, e.g., ClpB, belong to a group of Clp proteins, and contribute to protein folding, both during and after the translation process; moreover, they assist in protein secretion, avoid aggregation of proteins during heat shock, and repair damaged or misfolded proteins by stress action as a heat shock (Wawrzynow et al., 1996; Lund, 2001). The Clp machinery is extremely important for general protein turnover in LAB (Papadimitriou et al., 2016). GroEL and its co-chaperone GroES, act together in the correct folding of proteins mainly under stress conditions (Papadimitriou et al., 2016). Interestingly, the induction of these proteins was observed commonly in studies that evaluated the functional genome of LAB under conditions found by these bacteria mainly during the industrial processes. Transcriptomic analysis of *L. lactis* subsp. *cremoris* strains under conditions that mimic cheddar cheese manufacture show the induction of DnaJ and GroEL (Taïbi et al., 2011). A proteomic study conducted with *L. lactis* MG1363 show the induction of GroEL and GroES after heat and salt stress (Kilstrup et al., 1997). In *Lb. paracasei* NFBC 338, the *groESL* operon plays a key role in its viability during the drying process (Corcoran et al., 2006). The induction of these proteins at 37°C suggest that these chaperones might contribute in the NCDO 2118 adaptation under heat stress conditions preventing the possible deleterious aggregation of denatured proteins.

DNA lesion is commonly found in *L. lactis* under stress conditions, which cause a high genetic instability. Proteins related to DNA repair such as Phosphohydrolase, MutT/nudix and RmuC were more induced in NAE at 37°C. MutT acts in the prevention of errors in DNA replication by hydrolyzing mutagenic nucleotide substrates as 8-oxo-dGTP (Hamm et al., 2016), while, RmuC is part of the *rmuABC* loci, which prevents DNA inversions at short inverted repeats (Slupska et al., 2000). Moreover, uracil-DNA glycosylase was less induced in NAE (37°C: 30°C); this enzyme hydrolyzes the *N*-glycosidic bond to remove uracil in DNA and initiates the base excision repair process (Schormann et al., 2014). In the exclusive proteome of AE condition, we detected the 6-*O*-methylguanine-DNA methyltransferase (MGMT), which protects the DNA against alkyl groups as well as spontaneous G:C to A:T transitions (Aquilina et al., 1992).

### Transcription

Within the transcription category, some transcriptional regulators were less induced. The LysR was less induced in NAE (37°C: 30°C) and was detected in the AN exclusive proteome, this family of transcriptional regulators are highly conserved in prokaryotes and can act as activator or repressor of genes related to diverse processes such as metabolism, cell division, quorum sensing, virulence, motility, nitrogen fixation, oxidative stress, toxin production, attachment and secretion (Maddocks and Oyston, 2008). Another transcriptional regulator that was also less induced in this condition is MarR (multiple antibiotic resistance regulators). A study performed with *E. coli* showed that a gene related to antibiotic resistance is regulated by MarR (George and Levy, 1983). In addition, prior studies reported that members of the MarR family regulate genes related to different biological processes and are also involved in bacterial virulence (Ellison and Miller, 2006). The PhoU protein was detected as less induced in AN condition at 37°C; this protein acts as a negative regulator of the *pho* regulon, which is involved in phosphate transport (Steed and Wanner, 1993). In *E. coli*, besides participating in the regulation of phosphate transport, PhoU is involved in the regulation of genes related to cellular metabolism, antibiotic and stress resistance (Li and Zhang, 2007). Interestingly, a study shows that inactivation of this phosphate transporter contributes to the resistance process of *L. lactis* to oxidative stress (Cesselin et al., 2009). These identified transcription factors might act as the first line of sensors that contribute to the response of NCDO 2118 when exposed to these studied stress conditions.

### Proteins Involved in Cell Wall and Membrane Biogenesis

The components of the bacterial cell wall are described as important factors that contribute to the probiotic effect of different bacteria, due to its ability to stimulate the host immunomodulation process and promote an intestinal barrier against pathogens (Bron et al., 2011). Fibronectin is a component of the extracellular matrix (ECM) of epithelial cells; this glycoprotein is a target for bacterial adhesion in the gastrointestinal tract. This adhesion process is mediated by fibronectin-binding proteins expressed by both pathogens and commensal bacteria. We detected the fibronectin-binding protein 2A (FnBP2A) to be less induced in NAE (37°C: 30°C). FnBP are described in different probiotic bacteria such as: *Lb. acidophilus*, *Lb. casei*, *Lb. plantarum*, *Lb. brevis*, *Lb. rhamnosus*, and *Bacillus subtilis* (Hymes and Klaenhammer, 2016). A study showed that FnBPA from *Staphylococcus aureus*, when expressed at the surface of *L.* *lactis*, improved its immunomodulatory property in the context of vectors for DNA delivery (Almeida et al., 2016).

Penicillin-binding protein 2A (PBP2a) was less induced in NAE (37°C: 30°C), the PBPs are responsible for the transport of the *n*-acetyl-glucosamine (GlcNAc) through the plasma membrane and subsequent incorporation into peptidoglycan. In *S. aureus*, PBP2a was shown to be required for its ability to resist the action of beta-lactam antibiotic (Fuda et al., 2004). Interestingly, a study showed that PBP2a contributes to both intestinal stress robustness as well as resistance to acid and osmotic stress of *Lb. plantarum* WCFS1 (van Bokhorst-van de Veen et al., 2012). In addition, we also detected other proteins that contribute to the structure and peptidoglycan biosynthesis in the differentially expressed proteome as well as in the exclusive proteome, such as: family 2 glycosyltransferase, oligosaccharide glycerophosphotransferase and alanine racemase were more induced in NAE at 37°C. In AN condition, glycosyltransferase was more induced, while in the exclusive proteome this condition was detected for the following proteins: glucosyl transferase family 2, UDP-*N*-acetylglucosamine 2-epimerase and 1,4-beta-*N*-acetylmuramidase. The cell wall is required for shape as well as contributes to maintain the osmotic pressure. Additionally, the cellular envelope is described as the first line of defense against environmental stress. Thus, these proteins might have an important play role in maintaining the integrity of the cell wall, contributing to NCDO 2118 robustness to environmental stress.

### Proteins Involved in Metabolism

The majority of the proteins identified in both NAE and AN conditions were related to cellular metabolism. KEGG enrichment analysis showed difference between the metabolic pathways of NAE and AN conditions, which might reflect in the capacity of NCDO 2118 in modulating its metabolism in response to environmental changes. Concerning the nucleotide metabolism in NAE (37°C: 30°C) condition, our results show that NCDO 2118 reduce the *de novo* pyrimidine and purine nucleotide synthesis when exposed to 37°C. We detected the proteins PyrR, PyrP, PyrB, and CarA as less induced. The gene that codify these proteins are organized in an operon that controls the expression of genes involved in the pyrimidine biosynthesis, which lead to uridine monophosphate (UMP) formation (Martinussen et al., 2001). PyrR is described as a regulatory protein, while PyrP encodes a uracil permease transport, *pyrB*, encoding an aspartate transcarbamoylase, and *carA*, encoding the small subunit of the carbamoyl-phosphate synthetase. Other Pyr proteins such as PyrE and PyrF were also detected. In relation to purine metabolism, we detected the *pur* operon repressor (PurR), PurC, PurL and monophosphate dehydrogenase (GuaB) to be less induced in NAE at 37°C and ribose-phosphate pyrophosphokinase (Prs3) induced in AN (37°C: 30°C) condition. These proteins are involved in the purine *de novo* biosynthesis; this pathway starts with the conversion of phosphoribosyl pyrophosphate (PRPP) to inosine monophosphate (IMP) and consequently is modified to either Adenosine 5′-monophosphate (AMP) or Guanosine 5′-phosphate (GMP). *L. lactis* nucleotide metabolism, besides serving as substrates for RNA and DNA synthesis, is also described as a pathway that contributes to the resistance of this bacterium to different stress conditions (Duwat et al., 1999; Ryssel et al., 2014; Larsen et al., 2016).

Aldose 1-epimerase (GalM) and galactokinase (GalK) were detected as more abundant in both AN and NAE conditions at 37°C. These proteins are localized in the operon *galPMKTE*, which is involved in the metabolism of galactose via the Leloir Pathway (Grossiord et al., 2003). GalK is required for the fermentation process of galactose in *L. lactis* (Grossiord et al., 2003). GalM acts as a catalyzer in the interconversion process of the α- and β-anomers of galactose and is required for rapid lactose metabolism (Poolman et al., 1990; Bouffard et al., 1994). In addition, we detected differentially expressed proteins responsible for carbohydrate transport in both conditions such as: PTS sugar transporter, ABC transporter substrate-binding protein, sugar ABC transporter ATP-binding protein, PTS sucrose transporter subunit IIABC. In our proteomic analysis the N-acetylmannosamine-6-phosphate 2-epimerase (NanE) was more induced in AN (37°C: 30°C). In a transcriptomic study, this protein was related to amino sugar metabolism and was also induced in *L. lactis* upon exposure to heat stress (Dijkstra et al., 2016).

Proteins involved in amino acid metabolism were also modulated in NCDO 2118 upon exposition to NAE and AN conditions. Amino acid metabolism plays an important role in LAB physiology such as intracellular pH control, generation of metabolic energy or redox power, and stress resistance (Fernández and Zúñiga, 2006). Anthranilate synthase component I involved in the tryptophan biosynthesis was induced in AN condition at 37°C. Already, in NAE, Homoserine dehydrogenase was less induced at 37 °C; this enzyme catalyzes the reversible conversion of L-Aspartate 4-semialdehyde to L-homoserine. In addition, proteins such as Glutamine synthetase (GlnA), carbamoyl phosphate synthase large subunit (CarB), and carbamoyl phosphate synthase small subunit (CarA) were less induced at 37°C. These proteins are involved in the biosynthesis of carbamoyl phosphate from glutamate. Carbamoyl phosphate is an important metabolite that participates in nitrogen metabolism; it is a precursor of both arginine and pyrimidine. As described above pyrimidine metabolism was reduced at 37°C under NAE condition. On the other hand, ornithine carbamoyltransferase (ArcB) involved in the arginine deiminase pathway from carbamoyl phosphate was more induced in NAE at 37°C. This pathway provides ATP for bacterial growth in different environments, besides also being involved in the adaptation process of LAB to acid environments (Marquis et al., 1987; Casiano-Colón and Marquis, 1998).

Differences in the pyruvate metabolism was also observed between AN and NAE conditions. We detected the pyruvate formate lyase (PLF) and alcohol dehydrogenase enzyme (AdhE) in the exclusive proteome of AN condition; studies showed that both proteins are sensitive to oxygen (Takahashi et al., 1982; Arnau et al., 1998). Here, pyruvate is converted into acetyl coenzyme A (CoA) through PFL, and posteriorly AdhE metabolizes acetyl-CoA to acetaldehyde and ethanol. PLF acts mainly in the shift from homolactic to mixed-acid product formation during the *L. lactis* growth under anaerobic condition. This protein maintains an equimolar partitioning between acetate and ethanol generating an increase in ATP gain in relation to homolactic metabolism, being an alternative pathway utilized by *L. lactis* for pyruvate metabolism (Cocaign-Bousquet et al., 2002; Melchiorsen et al., 2002). In turn, in NAE (37°C: 30°C) condition, the pyruvate dehydrogenase E1 subunit alpha (PdhA) was detected as more induced; this protein is localized in the *pdhABC*. This complex is responsible for decarboxylation of pyruvate to acetyl coenzyme A (acetyl-CoA), which consequently gets processed in the tricarboxylic acid (TCA) cycle (Eikmanns and Blombach, 2014). In addition, acetate kinase (ACK) was also detected in NAE condition among the proteins with significant statistical significance. This enzyme is responsible for transforming Acetyl-CoA to acetate. Other studies also showed the difference in the pyruvate metabolism of *L. lactis* under anaerobic and micro aeration conditions (Jensen et al., 2001; Nordkvist et al., 2003). Jensen et al. (2001) showed that PFL and PDH activity are affected by oxygen bioavailability, which was also observed in our study. In addition, our results show that PDH is more active at 37°C in NAE condition.

**FIGURE 6:**
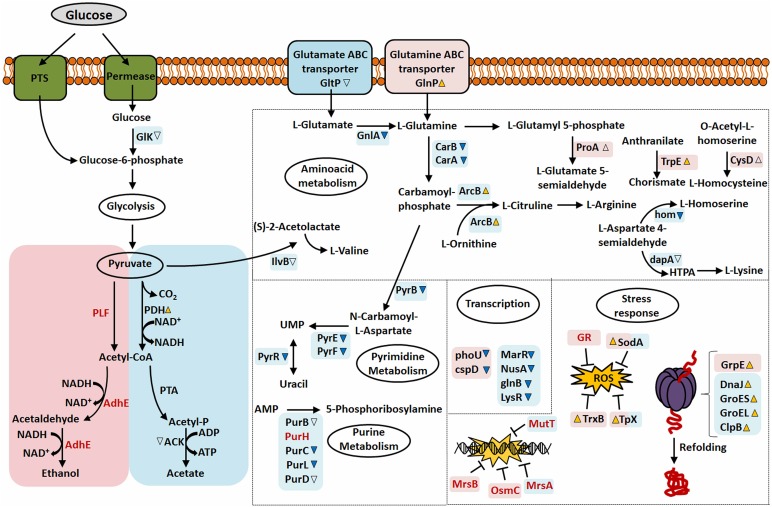
Overview of the differentially expressed, exclusive proteins and global response of NCDO 2118 to stress conditions studied. Pink or Blue squares correspond to proteins or pathways identified in AN and NAE condition, respectively. Differential expression of proteins (37°C: 30°C) with significant value increased or decreased (*p* < 0.05, cut-off values > 2-fold change) was depicted by yellow-up or blue-down arrows, respectively, while empty arrows represent proteins with *p* < 0.05 (cut-off values < 2-fold change). Squares that share pink and blue colors correspond to proteins that were identified as differentially expressed in NAE and AN conditions. Exclusive proteins are presented as red fonts.

Finally, based on the results obtained in our proteomic analysis, we show in Figure 6 the overview of the major mechanisms involved in the NCDO 2118 response to the stress conditions considered here.

### Identification of Strain-Specific Proteins

Studies have shown that probiotic activities generated by bacteria during the probiotic therapy are associated with strain-specific factors (Hungin et al., 2013; Sanders et al., 2013). In order to evaluate whether the proteins identified in our proteomic analysis could be strain-specific of NCDO 2118, we used the orthomcl tool (Li L. et al., 2003) to determine the pan-genome subsets with the core genes and the strain specific genes. This analysis revealed six proteins with unknown function that were predicted as exclusive to NCDO 2118 (Supplementary File S2, Table S9). The amino acid sequences of these proteins are available at Supplementary File S7. Among them, four proteins (NCDO2118_RS04930, NCDO2118_RS07360, NCDO2118_RS08490, NCDO2118_RS08730) were detected in the NAE and AN core proteome, and NCDO2118_RS08735 and NCDO2118_RS08645 were detected in the exclusive proteome of NAE and AN, respectively. Bioinformatics analyses were performed to identify possible subcellular localization and function. According to SurfG tool (Barinov et al., 2009), all proteins did not present positive predictions for signal peptides or trans-membrane domains and were predicted to be cytoplasmic. However, Secretome P (Bendtsen et al., 2005) analysis showed that the proteins NCDO2118_RS07360, NCDO2118_RS08645 and NCDO2118_RS08735 presented High SecP scores above 0.5, suggesting that these proteins might eventually be exported by non-classical secretion systems. The amino acid sequences of these proteins were BLASTed against the NCBI’s Conserved Domain Database (CDD) (Marchler-Bauer et al., 2017). Only in NCDO2118_RS08490 and NCDO2118_RS08645, we detected conserved domain sequence. In NCDO2118_RS08490 we detected the DUF3800 domain; the proteins in this family are described as functionally uncharacterized. The NCDO2118_RS08645 has two domains conserved: (i) Pleckstrin homology-like (PH-like): proteins with this domain are related to different functions, such as ability to bind inositol phosphates; (ii) Cyt_C5_DNA_methylase: this domain plays an important role in both DNA repair and genome stability. The identification of these strain-specific proteins motivates future studies to evaluate and explore the true role of each protein in NCDO 2118 physiology.

## Conclusion

In this work we applied the label-free proteomics approach to quantify the *L. lactis* subsp. *lactis* NCDO 2118 proteome in response to NAE or AN conditions under different temperatures. This comparative proteomic analysis generates different proteomic profiles which enabled the validation of *in silico* data of the *L. lactis* NCDO 2118 genome. The quantitative changes detected in our proteomic analysis showed the versatility of the functional genome of NCDO 2118, which reflects in its physiological adaptation to environmental changes that might be found during its passage inside TGI or in industrial processes. Altogether, these findings will help to improve our understanding about the molecular basis of the physiology of *L. lactis* NCDO 2118 as well as the robustness of this strain in response to stress conditions.

## Author Contributions

WM, HF, and VA conceived and designed the experiments. WM, CS, and GT performed microbiological analyses and sample preparation for proteomic analysis. WM, GT, and CR conducted the proteomic analysis. LO, SS, and FP performed bioinformatics analysis. PG, HF, and VA contributed substantially to data interpretation and revisions. WM, HF, and VA participated in all steps of the project as coordinators, and critically reviewed the manuscript. All authors read and approved the final manuscript.

## Conflict of Interest Statement

The authors declare that the research was conducted in the absence of any commercial or financial relationships that could be construed as a potential conflict of interest.

## Supplementary Material

FILE S1 (IMAGE 1) Growth curve of NCDO 2118 under AN (30°C and 37°C) and NAE (30°C and 37°C) conditions.

FILE S2 (TABLE 1) Supplementary Tables S1–S9.

FILE S3 (TABLE 2) Total list of peptide and proteins identified in AN condition.

FILE S4 (TABLE 3) Total list of peptide and proteins identified in AN condition.

FILE S5 (TABLE 4) Total list of peptide and proteins identified in NAE conditions.

FILE S6 (TABLE 5) Total list of peptide and proteins identified in NAE conditions.

FILE S7 Fasta sequence of strain-specific proteins of NCDO 2118 identified by LC-MSE analysis.
