# Supplementary figures and images for: Quantitative Proteomic Analysis of the Response of Probiotic Putative Lactococcus lactis NCDO 2118 Strain to Different Oxygen Availability Under Temperature Variation
Source: Front Microbiol. 2019 Apr 11;10:759. doi: 10.3389/fmicb.2019.00759 (PMC6470185; doi:10.3389/fmicb.2019.00759)

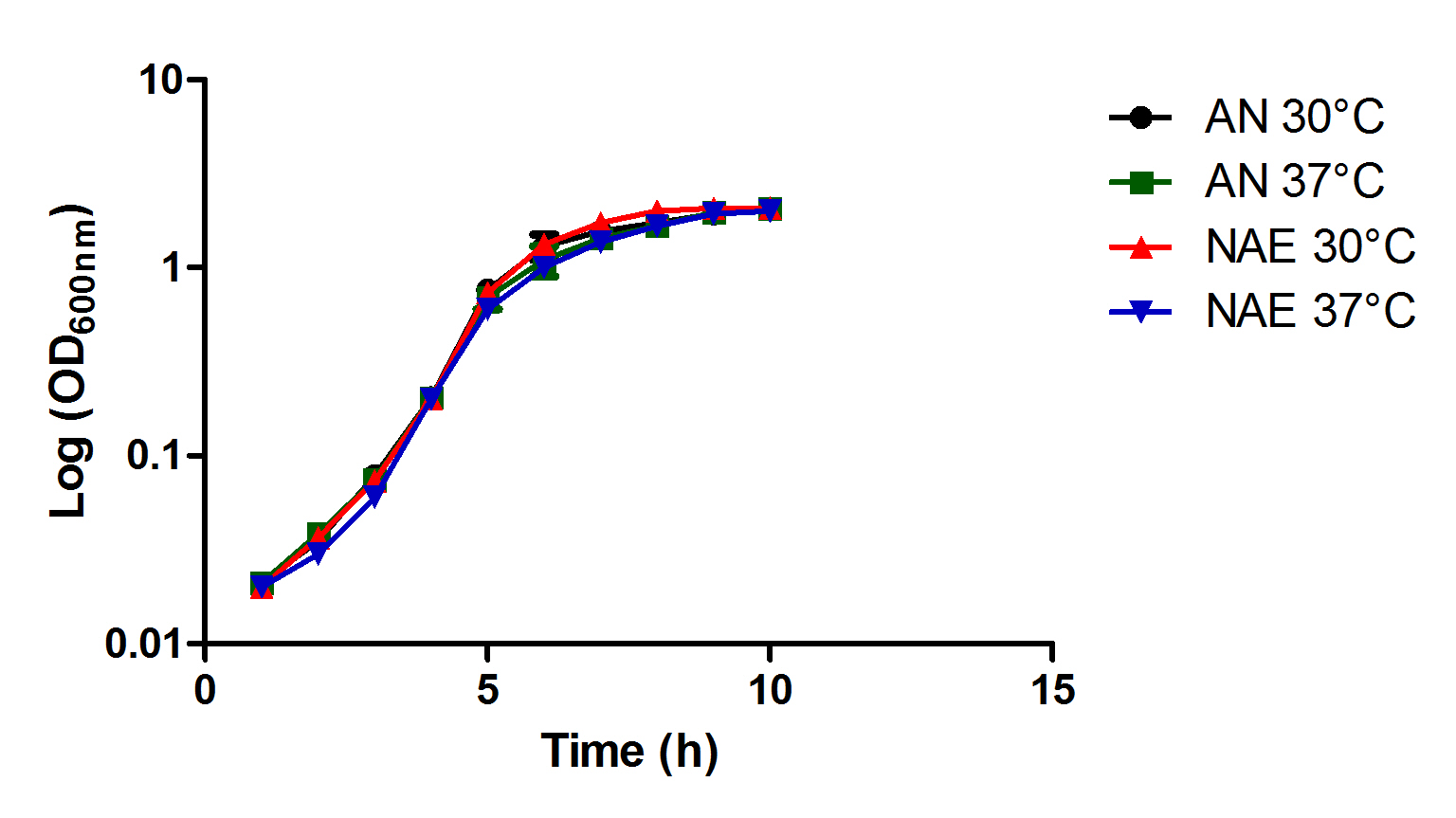

Supplement: FILE S1 (IMAGE 1) — Growth curve of NCDO 2118 under AN (30°C and 37°C) and NAE (30°C and 37°C) conditions. [file Image_1.JPEG]
