# Supplementary material for: Quantitative Proteomic Analysis of the Response of Probiotic Putative Lactococcus lactis NCDO 2118 Strain to Different Oxygen Availability Under Temperature Variation
Source: Front Microbiol. 2019 Apr 11;10:759. doi: 10.3389/fmicb.2019.00759 (PMC6470185; doi:10.3389/fmicb.2019.00759)
Supplement: FILE S7 — Fasta sequence of strain-specific proteins of NCDO 2118 identified by LC-MSE analysis. [file Data_Sheet_1.pdf]

File S.7: Fasta sequence of strain-specific proteins of NCDO 2118 identified by LC-MS^{E} analysis.

>NCDO2118_RS04930 NCDO2118_RS04930 hypothetical protein 1000763:1001092 reverse MW:12688

VDDIQLTKDSKQLLAIIYKEYLSKINNGVSKNSAKRIGHISDICELVPDWLPDDVLETMN

ELSRSEYVFNRYGNNTIIDSYLLDKTIIYFENKNINTVKEIADWVFKLI

>NCDO2118_RS07360 NCDO2118_RS07360 hypothetical protein 1537567:1538367 reverse MW:29298

IGAVAVLIIGGVSFALVKHNETGGVKTNASGLKSSSKKEMTSKTSEINESQKSSSSQTSS

SSQEETTQSQLSVDTKNLTENQFEQWVAITQSNQMQDTHPLRITVYKSDNNLHQAKIDYT

ATRIDSVNSYKVNSSGELEAIDFEHYPAMKVVSQSFPEIKQNQLTTNQINMWVVAVQDAL

FEQKGLKPGIDVVYDLETATDGNIANVKVFKIDYTFSANGGADNANKTLINEFRINSDGN

LEIKDVNSETGYRIISKVYMDTSMVQ

>NCDO2118_RS08490 NCDO2118_RS08490 hypothetical protein 1775121:1775813 reverse MW:26900

VQHVYFYFDDSGVLHPNAPERYFVYAGYVFIGKESKDDAIRKYRAVCQKIRDEIKSDGEI

KAFGLSNRNKNRLYQILAKEESVGLTVDIERVYSSILDNKKSIHRYKDYVLKMLVKEKLK

ELIFEQKINPNGSVALHIFVDEQPTSTDGFYKLRDTIYEEVKVGITNFQYNKRHAPLFNS

SVKCDVTFVDSSKYPLVQASDILANRIWNSFRLKKPNLREFVNHKCLHMP

>NCDO2118_RS08730 NCDO2118_RS08730 hypothetical protein 1810039:1810539 forward MW:19075

MNKLEITSFEYAVAVLNELASNNNESFVPFEIVWNTTLGPAKARTIIYDGTTPIISETII

GSDYIDRVFYNPRIKESDIFSFIRHEIFGYFEDKFSGLQHKSLIERHDLLMIKLLELSKL

DITSELTTPNYSTIFDFELIDGSMKLPFIHSDSIEIKEPISLISKN

>NCDO2118_RS08735 NCDO2118_RS08735 hypothetical protein 1810517:1810765 reverse MW:9754

MNPEDVKVTKESEEETARDRYLKNFKRDNLPLDFFGKPKLNEVGLKILDLLKKEDLTHEQ

AYASLQYVYNLIKYESNFLKLN

>NCDO2118_RS08645 NCDO2118_RS08645 hypothetical protein 1801600:1802301 reverse MW:27406

IDIENKNTDFLNLDLSDTSEYFGESNLFKELDKLPKPDIILASPPCESWSNASAMLNGNV

CWYTESTDTMFGQEFVSNEFTIRTRQQLETKNDTPFKKHWWKTVYSRLNGELCAFNTIRI

IERYQPEVWVIENPQSSRIWKYYKQIQDFQGIKNIAHYSAYDSERYSKKPTCFYSNLMFN

LKTTDEQSKLTFQGLGDKGISRSYNVRSEIPLQLIKDILDQCFLKLEKNSKEI
